# Commentary: sVEGFR1 Is Enriched in Hepatic Vein Blood—Evidence for a Provisional Hepatic Factor Candidate?

**DOI:** 10.3389/fped.2021.782779

**Published:** 2021-11-08

**Authors:** Carolina Putotto, Bruno Marino, Paolo Versacci

**Affiliations:** Department of Pediatrics, Obstetrics and Gynecology, “Sapienza” University of Rome, Rome, Italy

**Keywords:** congenital heart disease, Fontan operation, pulmonary arteriovenous malformation (PAVM), left isomerism, azygos continuation

## Introduction

Pulmonary arteriovenous malformations (PAVMs) represent a common complication of the bidirectional cavopulmonary anastomosis (Glenn) performed as first-stage surgical palliation for univentricular congenital heart disease. In the Glenn shunt, a preparatory procedure for Fontan operation, the superior vena cava blood flow is directed to the pulmonary arteries while the hepatic venous blood enters the right atrium and is excluded from the pulmonary blood flow. Although the lack of hepatic venous effluent directly perfusing the pulmonary arteries and the loss of pulsatile pulmonary blood flow have been identified as major etiological factors contributing to the development of PAVMs, their pathogenetic mechanism still remains unclear (1). Spearman et al. (2) suggested that soluble vascular endothelial growth factor receptor 1 (sVEGFR1) could be a potential hepatic factor candidate involved in the pathogenesis of PAVMs after surgical palliation for patients with univentricular heart.

## Relevant Subsection

In addition to patients undergoing univentricular palliation, PAVMs could be spontaneously present also in some children with heterotaxy, polysplenia, and left isomerism of the atrial appendages, although they have never undergone surgical treatment (3–7). In all of these previously reported patients, there was an interruption of the hepatic portion of the inferior vena cava with azygos continuation to superior vena cava and spontaneous pulmonary arteriovenous fistulas (3–7). This peculiar congenital pattern of systemic venous drainage could be associated with evident or “cryptic” intrahepatic disease. This combination of defects can result in an increased intrapulmonary shunting (8, 9) also without any surgical intervention (3–7). It is well-known that after Fontan repair with hepatic vein inclusion, PAVMs can completely regress. Anyway, Kim et al. demonstrated that in some patients who underwent Fontan completion, the PAVMs only partially resolved. All of them had left isomerism with azygos continuation of the inferior vena cava and the hepatic vein conduit on the contralateral side to the superior vena cava receiving azygos drainage. This would result in an imbalance of hepatic venous blood perfusion to the lungs bilaterally, which could lead to persistent PAVMs (10).

## Conclusion

In conclusion, the study conducted by Spearman et al. identified VEGFR1 as a possible factor implicated in the pulmonary microvascular remodeling and therefore potentially involved in the development of PAVMs in patients with palliated univentricular heart. However, also patients without univentricular heart but with the same abnormal hepatic venous flow distribution could develop PAVMs. So, these patients might profit from surgical redirection of hepatic venous blood into the pulmonary arteries.

Further studies should focus on detecting hepatic factor candidates in order to improve pathogenetic insights and find possible treatment strategies.

## Author Contributions

All authors wrote and critically revised the manuscript.

## Conflict of Interest

The authors declare that the research was conducted in the absence of any commercial or financial relationships that could be construed as a potential conflict of interest.

## Publisher's Note

All claims expressed in this article are solely those of the authors and do not necessarily represent those of their affiliated organizations, or those of the publisher, the editors and the reviewers. Any product that may be evaluated in this article, or claim that may be made by its manufacturer, is not guaranteed or endorsed by the publisher.

## References

[1] Kavarana MN Jones JA Stroud RE Bradley SM Ikonomidis JS Mukherjee R. Pulmonary arteriovenous malformations after the superior cavopulmonary shunt: mechanisms and clinical implications. Expert Rev Cardiovasc Ther. (2014) 12:703–13. 10.1586/14779072.2014.912132 24758411 PMC4410694

[2] Spearman AD Gupta A Pan AY Gudausky TM Foerster SR Konduri GG. sVEGFR1 is enriched in hepatic vein blood-evidence for a provisional hepatic factor candidate? Front Pediatr. (2021) 9:679572. 10.3389/fped.2021.679572 34195162 PMC8236596

[3] Papagiannis J Kanter RJ Effman EL Pratt PC Marcille R Browning IB 3rd. Polysplenia with pulmonary arteriovenous malformations. Pediatr Cardiol. (1993) 14:127–9. 10.1007/BF00796995 8469631

[4] Amodeo A Di Donato R Carotti A Marino B Marcelletti C. Pulmonary arteriovenous fistulas and polysplenia syndrome. J Thorac Cardiovasc Surg. (1994) 107:1378–9. 10.1016/S0022-5223(94)70076-1 8176990

[5] Srivastava D Preminger T Lock JE Mandell V Keane JF Mayer JE Jr. Hepatic venous blood and the development of pulmonary arteriovenous malformations in congenital heart disease. Circulation. (1995) 92:1217–22. 10.1161/01.CIR.92.5.1217 7648668

[6] Kawata H Kishimoto H Ikawa S Ueno T Nakajima T Kayatani F. Pulmonary and systemic arteriovenous fistulas in patients with left isomerism. Cardiol Young. (1998) 8:290–4. 10.1017/S1047951100006788 9731642

[7] Amodeo A Marino B. Pulmonary arteriovenous fistulas in patients with left isomerism and cardiac malformations. Cardiol Young. (1998) 8:283–4. 10.1017/S1047951100006752 9731639

[8] Fewtrell MS Noble-Jamieson G Revell S Valente J Friend P Johnston P. Intrapulmonary shunting in the biliary atresia/polysplenia syndrome: reversal after liver transplantation. Arch Dis Child. (1994) 70:501–4. 10.1136/adc.70.6.501 8048820 PMC1029869

[9] Barbé T Losay J Grimon G Devictor D Sardet A Gauthier F. Pulmonary arteriovenous shunting in children with liver disease. J Pediatr. (1995) 126:571–9. 10.1016/S0022-3476(95)70351-9 7699535

[10] Kim SJ Bae EJ Lee JY Lim HG Lee C Lee CH. Inclusion of hepatic venous drainage in patients with pulmonary arteriovenous fistulas. Ann Thorac Surg. (2009) 87:548–53. 10.1016/j.athoracsur.2008.10.024 19161777

